# Modern peptide biomarkers and echocardiography in cardiac healthy haemodialysis patients

**DOI:** 10.1186/s12882-017-0589-3

**Published:** 2017-05-30

**Authors:** Franz Maximilian Rasche, Stephan Stoebe, Thomas Ebert, Silvana Feige, Andreas Hagendorff, Wilma Gertrud Rasche, Filip Barinka, Volker Busch, Ulrich Sack, Jochen G. Schneider, Stephan Schiekofer

**Affiliations:** 1 Department of Internal Medicine, Neurology, Dermatology, Clinic for Endocrinology, Diabetology and Nephrology, Section of Nephrology, University Leipzig, Leipzig, Germany; 2 Department of Internal Medicine, Neurology, Dermatology, Clinic for Cardiology and Angiology, University Leipzig, Leipzig, Germany; 3 Department of Head Medicine and Oral Health, Department of Ophthalmology, University Leipzig, Leipzig, Germany; 4 Center for Geriatric Medicine at Bezirksklinikum Regensburg, Universitätsstr. 84, 93053 Regensburg, Germany; 5 Institute of Clinical Immunology, Medical Faculty, University of Leipzig, Leipzig, Germany; 6 Translational & Experimental Medicine, Luxembourg Centre de Systems Biomedicine, Luxembourg City, Luxembourg; 7 Department of Internal Medicine II, Saarland University, Homburg Saar, Germany

**Keywords:** Haemodialysis, Copeptin, AVP, MR-proANP, NT-proBNP, Cardiorenal syndrome, Left ventricular dysfunction

## Abstract

**Background:**

In this prospective study, we aimed to assess the haemodynamic changes before and after haemodialysis (HD) in cardiac healthy subjects on chronic HD by imaging methods and endocrine markers of fluid balance.

**Methods:**

Mid-regional pro-atrial natriuretic peptide (MR-proANP), N-terminal prohormone of brain natriuretic peptide (NT-proBNP), vasopressin (AVP) and copeptin (CT-proAVP), metanephrines and normetanephrines, renin and aldosterone, standard transthoracic echocardiography and diameter of vena cava inferior (VCID) were performed in 20 patients with end stage renal disease (CKD5D) before and after HD and were stratified in residual excretion (RE, less or more 0.5 l) and ultrafiltration rate (UF, less or more 2 l).

**Results:**

Copeptin was significantly higher in patients before HD. Copeptin was inversely correlated with haemodialysis treatment adequacy (KT/v), RE and UF, but was not significantly influenced by age, gender and body mass index (BMI). MR-proANP was significantly reduced by haemodialysis by 27% and was inversely correlated with KT/v, but there was a significant influence by UF, RE, age, gender and BMI. NT-proBNP was significantly higher in patients before HD and was not influenced by RE and UF. Renin, aldosterone, metanephrines and normetanephrines did not demonstrate significant differences. Echocardiographic parameters and VCID were significantly correlated with RE, UF and copeptin.

**Conclusion:**

Modern biomarkers will provide cardiovascular risk assessment, but elimination (UF), RE and other factors may influence the serum concentrations, e.g. in patients with renal impairment. The interpretation will be limited by altered reference ranges, and will be restricted to individual courses combined with clinical and echocardiographic data.

**Electronic supplementary material:**

The online version of this article (doi:10.1186/s12882-017-0589-3) contains supplementary material, which is available to authorized users.

## Background

Cardiovascular mortality in haemodialysis (HD) patients (CKD5D) is tenfold higher than in the normal population [1]. In patients with chronic kidney disease (CKD), excessive volume overload and arterial hypertension evoke characteristic morphological and functional cardiac changes – the chronic reno-cardiac syndrome type 4 (CRS) with left ventricular remodelling, dilatation of the left ventricle and the development of left ventricular hypertrophy [2–4]. Consequently, these patients are predisposed for the development of cardiac arrhythmias, uremic cardiomyopathy - a hybrid of hypertrophic and dilative cardiomyopathy - and finally left and right ventricular heart ailure [5–7]. Reliable serum biomarkers for prevention or monitoring of CRS are necessary in this high risk population in which morphological diagnostic is limited because of adverse effects of contrast media, e. g. contrast media nephropathy or nephrogenic fibrosis [8]. However, residual renal excretion and/or elimination by HD might have a significant influence on serum concentrations of these markers.

In the present study, we aimed to assess the influence of volume balance, residual kidney excretion, and elimination by HD. Therefore we measured novel biomarkers in cardiac healthy patients with CKD5D, e.g. copeptin [9–16], MR-proANP [17, 18], NT-proBNP [19–25] and non-invasive ultrasound parameters, i.e. oscillation of the diameter of the vena cava inferior and echocardiographic parameters, before and after HD.

## Methods

Twenty cardiac healthy patients (male *n* = 10, female *n* = 10; glomerulonephritis *n* = 8, obstructive nephropathy *n* = 4, polycystic kidney disease *n* = 1, diabetes mellitus *n* = 1, unknown = 6) with a median age of 48 years (mean 48; SD 9; range 29 to 62) and CKD5D (median time on HD 29 months; range 3 to 102) were included in the prospective study at our outpatient haemodialysis care unit. All clinical investigations were performed at the midweek HD session.

Exclusion criteria were severe malignancy, arterial hypertension, acute myocardial infarction, heart failure, structural heart disease, ascites, pericardial or pleural effusion, complex arrhythmias, and moderate or severe valvular heart disease, pacemakers or other devices. None of the patients suffered from peripheral artery disease, stroke or diabetes mellitus, except only one patient with diabetes mellitus type 1 with kidney and pancreas transplantation and with exclusion of ischemic heart disease by invasive diagnostics before renal transplantation. In this single patient, transplant function of the kidney was lost by histologically confirmed chronic glomerulopathy after transplantation without any signs of diabetes mellitus. However, the endocrine pancreas function still remained until these days with sufficient blood glucose control (HbA1c < 5.5%).

The study protocol was approved by the institutional review board, Medical Faculty of the University of Leipzig (No. 089-10-19,042,010), and informed consent was obtained from all patients.

### Haemodialysis prescriptions

Haemodialysis was performed three times per week with a mean time of 4.85 h (five hours *n* = 17; four hours *n* = 3), proven arterial fistula blood flow >800 ml/min, machine blood flow 300 ml/min, dialysate flow 500 ml/min, dialysate concentrations SW 285A (Na^+^ 138, K^+^ 3) and SW 393A (Na^+^ 138, K^+^ 4) (Braun, Germany), high flux membrane/dialyzer Polyflux 170H (Nikkiso, Japan), and haemodialysis machine DBB05 (Nikkiso, Japan). All patients had native fistulas. The cut off of the ultrafiltration rate was set by 2000 ml by the median of all patients of the employed session for the dichotomous variable “UF 2000 ml” (median 2000 ml, min 0 ml, max 5000 ml, mean 2055 ml).

### Biochemistry

Haemoglobin (Hb), haematocrit (Hk), sodium, potassium, phosphate, creatinine, osmolality, albumine, vasopressin (AVP), aldosterone, renin, metanephrine, normetanephrine, MR-proANP, and copeptin [26] have been obtained by serum and plasma samples (EDTA, citrate, and heparin) before and after haemodialysis. Due to intradialytic elimination, NT-proBNP was solely measured before haemodialysis [27]. Additionally, KT/v was calculated using the Daugirdas formula [28, 29].

### Echocardiography

In all patients standardised transthoracic echocardiography was performed by experienced cardiologists according to national and international recommendations before (after suitable rest of 20 min for each patient) and approximately 20 min after haemodialysis [30]. Echocardiographic investigations were performed with Vivid 7 or Vivid E9 (GE Healthcare, Frankfurt, Germany) and all parameters were analysed offline using the EchoPac-Software (GE Healthcare, Frankfurt, Germany).

Left ventricular (LV) systolic function was assessed by determining LV ejection fraction due to biplane LV volume analysis using the modified Simpson’s rule [31]. Left ventricular diastolic function was assessed by the following echocardiographic parameters. The early-diastolic (Emax) and late-diastolic (Amax) maximum velocity of the mitral valve inflow were assessed by pulsed-wave Doppler measurements in the apical long axis view. The sample volume was positioned 1-2 cm above the mitral ring at the junction of the mitral leaflets to the chord strands and inappropriate Doppler angles were avoided. Both peak velocities permit the calculation of the E/A-ratio. In addition, the deceleration time (EDT) describing the duration from E peak to the end of the E-wave.

The left ventricular end-diastolic pressure was assessed by the determination of E/E’-ratio. E’ describes the tissue velocity of the motion of a specific myocardial segment during diastole and was assessed by tissue Doppler measurements. The sample volume of the pulsed-wave tissue Doppler was positioned at the basal inferoseptal and basal lateral segment in the apical 4-chamber view to obtain E’-inferoseptal and E’-lateral, respectively. Thus, the E/E’-inferoseptal-ratio and E/E’-lateral-ratio could be determined [32].

The diameter of the vena cava inferior (VCID) was assessed in a subcostal short axis view where the vena cava inferior enters the right atrium. Three different diameters were determined via M-Mode during expiration of the patient to obtain the median of the VCID.

The echocardiographic parameters were correlated with the ultrafiltration rate (UR) and the residual excretion (RE).

### Statistical methods

Non-parametric tests to compare group values (Wilcoxon-test, Friedman test, and Mann-Whitney-U-test) and bivariate correlation analysis (Spearman test, r regression coefficient) were performed as indicated. Statistical significance for all tests was set at a level of *p* < 0.05. Statistical analysis was done using the SPSS version 21.0 software package (SPSS Inc., Chicago, Illinois, USA). If not indicated otherwise, data are given as median values with range (minimum to maximum), or mean and standard deviation (± SD).

## Results

### Pre- and post-HD sampling, ultrafiltration rate, and residual urinary excretion rate – Influence on serum risk markers

Pre- and post-HD sampling was performed in our study and biochemistry parameters were determined before and after HD as shown in Table 1.

**Table 1:** Biochemistry parameters before and after haemodialysis (HD)

Parameters	Before HD median (min - max)	After HD median (min - max)	Normal range^a^	Significance
Haemoglobin (g/dl)	7.2 (5.5-8.2)	7.4 (5.5-9.1)	7.6-9.5	*p* = 0.007
Haematokrit (%)	0.36 (0.28-0.41)	0.37 (0.27-0.44)	0.35-0.47	*p* = 0.229
Potassium (mmol/l)	5.3 (2-6.2)	4.2 (3.6-4.9)	3.3-5.4	*p* = 0.02
Sodium (mmol/l)	136 (133-147)	137 (132-140)	134-146	*p* = 0.168
Phosphate (mmol/l)	1.6 (0.8-3.3)	0.9 (0.4-1)	0.9-1.5	*p* < 0.001
Urea (mmol/l)	23 (10-37)	6 (3-15)	1.7-8.3	*p* < 0.001
Creatinine (μmol/l)	929 (182-1481)	357 (116-702)	< 80	*p* < 0.001
Albumin (g/l)	44.5 (37-48)	45 (35-52)	34-48	*p* = 0.764
Osmolality (mosm/kgH2O)	318 (301-338)	302 (291-326)	280-300	*p* < 0.001
Aldosterone (ng/l)	148 (35-615)	79 (10-707)	20-150	*p* = 0.191
Renin active (direct) (ng/l)	12 (1-683)	18 (0.6-1705)	1.68-23.9	*p* = 0.038
Metanephrine (free) (ng/l)	146 (54-349)	78 (32-308)	< 90	*p* = 0.001
Normetanephrine (free) (ng/l)	459 (92-822)	188 (75-328)	< 180	*p* < 0.001
AVP (ng/l)	7.6 (1-9.4)	5.1 (1-9.1)	< 8	*p* < 0.001
CT-proAVP (pmol/l)	159 (14-291)	63 (8-132)	1-14	*p* < 0.001
MR-proANP (pmol/l)	814 (285-2908)	665 (222-2144)	46.1-85.2	*p* < 0.001
NT-proBNP (ng/l)	3020 (522-72,312)	-	< 169	

*Abbreviations*: *HD* haemodialysis, *AVP* anti-diuretic hormone/vasopressin, *CT-proAVP* carboxy terminal pro-arginin-vasopressin (copeptin), *MR-proANP* midregional fragment of the N-terminal of pro-ANP, *NT-proBNP* N-terminal pro-brain natriuretic peptide, *BMI* body mass index, *K* dialyzer clearance of urea, *t* dialysis time, *V* volume of distribution of urea approximately equal to patient’s total body water, *URR* Urea reduction rate, *p* significance level in Wilcoxon-test

^a^normal range in healthy patients without HD

In patients with an ultrafiltration rate ≥ 2000 ml aldosterone post-HD was significantly higher as in patients with an ultrafiltration rate < 2000 ml (Additional file 1: Table S1). In patients with a residual urinary excretion rate < 500 ml normetanephrine pre-HD was significantly lower as in patients with a rate > 500 ml. Copeptin pre- and post-HD was significantly higher in patients with an urinary excretion rate < 500 ml (Additional file 1: Table S2).

### AVP and copeptin in regard to UF, RE, age, gender, BMI, and others

**Fig. 1:**
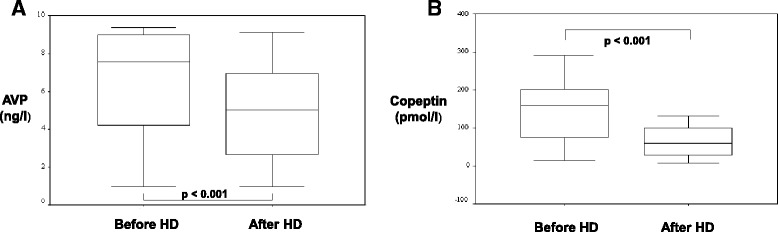
**a** AVP concentration before and after haemodialysis (HD). **b** Copeptin concentration before and after haemodialysis (HD)

**Fig. 2:**
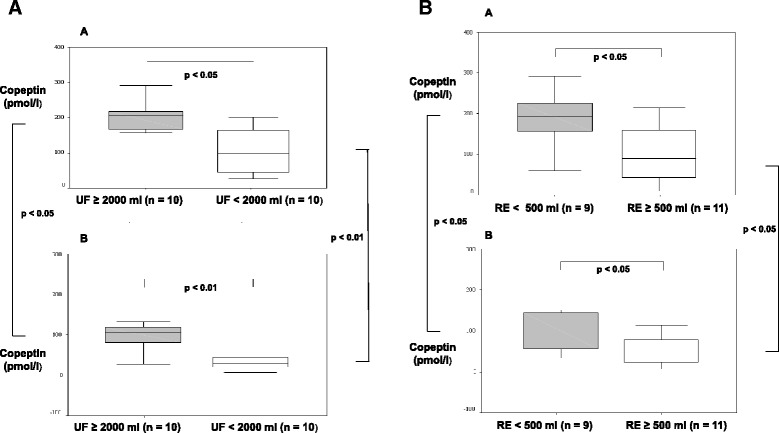
**a** Copeptin concentration before (*a*) and after (*b*) haemodialysis (HD) depending on ultrafiltration rate (UF). **b** Copeptin concentration before (*a*) and after (*b*) haemodialysis (HD) depending on patient residual excretion (RE)

AVP was in normal range before HD and decreased after HD significantly by 27% (Fig. 1a). Only the percental reduction of copeptin (and not of AVP) was significantly correlated with the percental reduction of the body weight after HD (Spearman test in bivariate correlation analysis, *p* = 0.028, *r* = −0.503). In patients with an UF > 2000 ml, serum AVP levels were significantly higher (before HD: median 8.9 ng/l (4.4 - 9.4 ng/l); after HD median 6.9 ng/l (2.9 - 9.1 ng/l) than in patients with an UF < 2000 ml (before HD median 5.1 ng/l (1 - 7.7 ng/l; after HD median 2.7 ng/l (1 - 6.1 ng/l); *p* < 0.05). There was a trend in higher AVP levels in patients with a RE ≥ 500 ml (Additional file 1: Table S2). No influence of gender, age, or BMI was found. Copeptin levels were 11 fold higher in patients with HD as in the normal population (median 159 pmol/l, 14 - 291 pmol/l, normal median 4.2 pmol/l, 1 - 14 pmol/l [26] and there was a significant reduction of 54% by HD (Fig. 1b). Copeptin levels were significantly higher in patients with an UF ≥ 2000 before HD (median 207 pmol/l, 59 - 291 pmol/l, *p* < 0.05) and after HD (median 110 pmol/, 27 - 132 pmol/l, *p* < 0.01) than in patients with an UF < 2000 (median before HD 90 pmol/l, 14 - 196 pmol/l; median after HD 29 pmol/l, 8 - 75 pmol/l). After HD there was in both groups a significant reduction of copeptin and copeptin was significantly correlated with UF (Fig. 2A). Copeptin values were also correlated with the body weight. Thus, in case of increased body weight loss - in case of higher UF - during HD higher copeptin values were obtained (Fig. 2B). Significantly higher copeptin values were revealed in the group with RE < 500 ml (*n* = 9), either pre-HD (Median 193 pmol/l; 59 - 291 pmol/l) or post-HD (Median 106 pmol/l; 27 - 132 pmol/l) in comparison to the study group with RE ≥ 500 ml (*n* = 11, median before HD 95 pmol/l, 14 - 218 pmol/l; median after HD 39, 8 - 114 pmol/l). In addition, the statistical analysis showed a negative correlation between copeptin and RE (data not shown). Interestingly, older, male and obese patients tended to higher copeptin concentrations. The relative reduction of copeptin during HD significantly correlated inversely with the Kt/V ratio and the urea reduction indicating that copeptin might be eliminated by HD.

### MR-proANP and NT-proBNP before and after HD

In parallel to AVP and copeptin, MR-proANP was significantly reduced about 26.8% after HD (median before HD 814 pmol/l (285 - 2908 pmol/l), median after HD 665 pmol/l (222 - 2144 pmol/l). Similarly to copeptin, all MR-proANP values were clearly above the normal range as described in the literature in non HD patients. Before HD, MR-proANP values were up to ten fold higher as in the normal population and after HD MR-proANP was still up to eight fold above the normal range in non HD patients (46.1 to 85.2 pmol/l). The percental reduction of MR-proANP was not significantly correlated with the percental reduction of the body weight after HD (Spearman test in bivariate correlation analysis, *p* = 0.9, *r* = −0.029). However, there was no significant difference relating to UF, RE, age, gender and BMI. In analogy to copeptin or MR-proANP, the NT-proBNP concentration (median 3020 ng/l, 522 - 72,312 ng/l) was about 18 fold elevated in comparison to the normal range (<169 ng/l).

### Renin

There was an increase of renin after HD in 13 patients and a decrease of renin in seven patients after HD. In addition, there was a positive correlation of renin to UF after HD showing that a higher UR rate led to higher renin values after HD (*r* = 0.5 and *p* < 0.05). The percental reduction of renin was significantly correlated with the percental reduction of the body weight after HD (Spearman test in bivariate correlation analysis, *p* = 0.046, *r* = −0.452). Other significant correlations between renin and e.g. RE, age, BMI, blood pressure, antihypertensive drugs or remaining biochemistry parameters as proANP, proBNP, copeptin, AVP, aldosterone, meta- and normetanephrines could not be revealed (data not shown).

According to the different renin reaction patterns, patients with renin decrease (*n* = 7) and with renin increase (*n* = 13) after HD showed both significant differences for renin. A comparison of parameters depending from post-HD renin decrease or renin increase is shown in Additional file 1: Table S3.

### Aldosterone

An inverse correlation between aldosterone and proANP was detected (*r* = −0.6, *p* < 0.01). This was similar to proBNP showing a significantly negative correlation to aldosterone (*r* = −0.5, *p* < 0.05). The percental reduction of aldosterone was significantly correlated with the percental reduction of the body weight after HD (Spearman test in bivariate correlation analysis, *p* = 0.00035, *r* = −0.790). Other significant correlations (e.g. age, BMI, gender, blood pressure, antihypertensive drugs or remaining biochemistry parameters) could not be revealed.

Similarly to renin, different aldosterone reaction patterns could be detected: patients with aldosterone decrease (*n* = 15) and with aldosterone increase (*n* = 5) after HD showed both significant differences for aldosterone. A comparison of parameters depending on post-HD aldosterone decrease (e. g. systolic blood pressure 2 h after the begin of HD) or aldosterone increase (e. g. haemoglobin, haematocrit, metanephrines) is shown in Additional file 1: Table S4.

### Metanephrines and normetanephrines

**Fig. 3:**
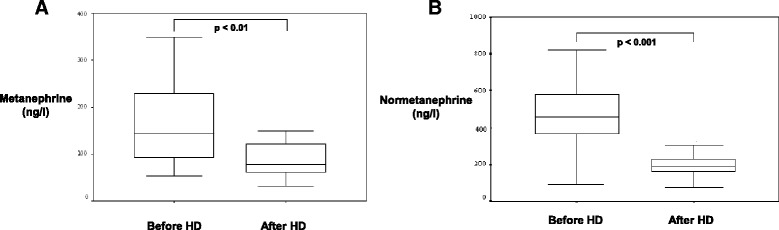
**a** Metanephrine concentration before and after haemodialysis (HD). **b** Normetanephrine concentration before and after haemodialysis (HD)

Pre-HD metanephrine values were generally above the normal range (<90 ng/l) for metanephrines and there was a significant decrease after HD (Fig. 3a).

Normetanephrine concentrations were pre-HD and post-HD elevated above the normal range (< 180 ng/l). A significant reduction of normetanephrines was detectable post-HD in comparison to pre-HD normetanephrine concentrations similarly to the metanephrines. The median decrease of normetanephrines with 56.1% was slightly higher compared to the metanephrine decrease (Fig. 3b). The percental reduction of metanephrines and normetanephrines was not significantly correlated with the percental reduction of the body weight after HD (Spearman test in bivariate correlation analysis, *p* = 0.887, *r* = −0.034, *p* = 0.572, *r* = − 0,134).

### Echocardiographic analysis of left ventricular dysfunction and determination of the vena cava inferior-diameter before and after haemodialysis

The echocardiographic data sets could be analysed in 15 of 20 patients in a standardized professional setting and methods [30, 32]. Three patients were excluded due to insufficient image quality and one patient each due to unknown mitral stenosis and atrial fibrillation. An overview of the echocardiographic parameters before and after HD is shown in Additional file 1: Table S5.

### Conventional parameters (Doppler echocardiography)

**Fig. 4:**
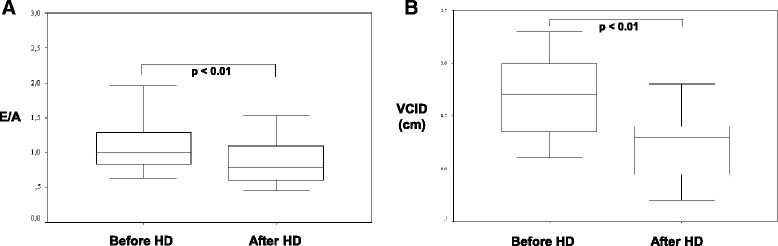
**a** E/A - ratio (early diastolic velocity of the mitral inflow/late-diastolic velocity of the mitral inflow - ratio) before and after haemodialysis (HD). **b** Vena cava inferior-diameter (VCID) before and after haemodialysis (HD)

The maximum early-diastolic (Emax, *p* > 0.05), maximum late-diastolic (Amax; *p* < 0.05) velocity and the E/A-ratio (*p* < 0.05) of the mitral valve inflow were lower after HD whereas Amax and the E/A-ratio were significantly reduced after HD (Additional file 1: Table S5 and Fig. 4a). TAPSE (tricuspid annular plane systolic excursion, *n* = 16) and SPAP (systolic pulmonary arterial pressure, *n* = 13) were routinely performed for the investigation of the right ventricular function. TAPSE was not significantly different before 2.20 cm (1.5 to 3.0) and after 2.25 cm (1.6 to 3.0) HD (Wilcoxon-test, *p* = 0.57) but SPAP was significantly reduced from 33.01 mmHg (19.17 to 72.92, SPAP >30, *n* = 11) to 29.27 mmHg (14.79 to 53.43, SPAP >30, *n* = 6, Wilcoxon-test, *p* = 0.0051). Only the SPAP after HD was significantly different in patients with an UF rate less or bigger 2000 ml per session (*p* = 0.018) and RE less or bigger 500 ml per day (Mann-Whitney-U-test, *p* = 0.005). TAPSE, SPAP, copeptin, renin and aldosterone before and after HD were not significantly correlated in absolute values and in the difference values (before and after HD) in the correlation analysis (Spearman test, *p* > 0.05).

### Tissue-Doppler imaging (TDI)

The maximum early-diastolic tissue velocity Vmax E’ in region of the basal inferoseptal left ventricular segment was not significantly reduced whereas the Vmax E’ in region of the basal lateral left ventricular segment was significantly reduced after HD (Additional file 1: Table S5). Significant changes in left ventricular end-diastolic pressure (E/E’ inferoseptal, E/E’ lateral) could not be observed after HD. However, values for Vmax E’ inferoseptal were lower than values for Vmax E’ lateral leading to an increased E/E’-ratio when the left ventricular pressure was estimated in region of the basal inferoseptal region of the left ventricle.

### Comparison of echocardiographic parameters depending on the ultrafiltration rate and the residual excretion

Concerning the comparison of the echocardiographic parameters depending on the UF the percentage reduction of the VCID was significantly higher in patients with an UF ≥ 2000 ml (*n* = 8) than in patients with an UF of <2000 ml (*n* = 7) after HD. The other parameters did not differ depending on the UF (Additional file 1: Table S6).

The comparison of the echocardiographic parameters depending on the RE showed lower maximum early-diastolic velocity values (Emax) in patients with a RE < 500 ml (*n* = 8) than in patients with a RE of ≥500 ml (*n* = 7) before HD. The other parameters did not differ depending on the RE (Additional file 1: Table S7).

### Copeptin and renin in relation to echocardiographic parameters

In addition, significant correlations between echocardiographic parameters and biochemistry parameters have been revealed before and after haemodialysis, e.g. copeptin and renin with Amax, E/E’ inferoseptal and lateral. These data are shown in Additional file 1: Table S8.

### Vena cava inferior-diameter

The VCID did show ranges between 13 and 28 mm before and after HD. VCID values of less than 13 mm could be observed in two patients before HD and in seven patients after HD (Fig. 4b). Interestingly, a positive correlation between VCID before HD and patient weights before and after HD was calculated. In addition, the percentage decrease of VCID correlated to UR and other biochemistry parameters (Additional file 1: Table S9). A significant correlation between VCID and echocardiographic parameters or patient body weights has not been revealed.

## Discussion

Overt or occult fluid overload is associated with higher cardiovascular mortality in CKD patients [14, 33–35]. Modern biomarkers for endocrine water homoeostasis, e. g. copeptin, identified in larger epidemiological studies in renal healthy subjects might be useful in the detection of fluid overload and prediction of cardiovascular mortality or outcome of sepsis (Additional file 1: Table S10) [26, 36–42]. Ideally, these markers should fulfill the following specifications and characteristics: *i.* easy accessible and no additional stress for the patient or nurses, *ii.* little pre-analytic effort and analyte stability, *iii.* no elimination by HD, *iv*. easy to adjust individual characteristics, e. g. residual renal excretion, gender, age, etc., and *v.* cost effectiveness. However, in CKD5D patients, large variability or deviation of modern peptide biomarker concentrations is related to several factors.

We have investigated the influence of residual kidney function and ultrafiltration in cardiac healthy CKD5D patients on parameters of fluid homeostasis in the vasopressin, sympathic- and the renin aldosterone system, in markers of the dilatation of the cardiac muscle and morphologic alterations in echocardiography and VCID. Copeptin is a 39-amino acid-long peptide derived from a pre-pro-hormone consisting of AVP, neurophysin II and copeptin. AVP is involved in multiple cardiovascular and renal pathways and functions. However, AVP measurements are not commonly used in clinical practice because of the biochemical features of the molecule. On the other hand, copeptin can be immunologically tested with ease and therefore be used as an AVP surrogate. Copeptin was significantly altered by the residual excretion and the ultrafiltration rate in our study but was not correlated with the renin-aldosterone system or the sympathetic nervous system. Therefore, copeptin is a new, promising reliable marker for fluid overload and cardiovascular mortality and is directly influenced by residual excretion and the ultrafiltration rate.

However, elimination by renal excretion and haemodialysis might be suspected for copeptin and individual values have to be interpreted with caution in analogy to other biomarkers [43–45]. It might be supposed that multiple sequential measurements focused on the individual variation could allow a prognostic value of this parameter in analogy to NT-proBNP [46]. On the other hand, classical biomarkers parameters did not correlated significantly with residual excretion or ultrafiltration in our study. Additionally, we have demonstrated no significant influence on the sympathetic nervous system and on the renin – aldosterone system.

Therefore, the ideal modern biomarker for the individual assessment of fluid overload could not be evaluated in this study which was analogue to other studies [4, 43, 45, 46]. Our study is limited to cardiac healthy patients and reflects the answer of the endocrine system and the heart function in one HD session. Therefore, future studies should also investigate other biomarkers employed in the cardiorenal syndrom, such as Cystatin C or TIMP – 2. However, a long term follow up of cardiac healthy and comprimised patients with the new biomarkers might contribute to risk management in these patients.

Interestingly, strong correlations were found with echocardiographic parameters and VCID (Additional file 1: Table S9 and S10). In general, inferoseptal E’ velocities were lower than lateral E’ velocities leading to higher inferoseptal E/E’ ratios which is analogue to former studies [32]. In addition, recent studies have shown, that in patients with normal ejection fraction lateral E/E’ have better correlations with left ventricular filling pressures, so that it could be assumed that lateral Vmax E’ and lateral E/E’ are more suitable for an echocardiographic analysis in healthy subjects. In the present study no significant differences of Vmax E’ or E/E’ obtained by measurements in the inferoseptal or lateral region could be observed. However, VCID seems to be the most suitable echocardiographic parameter for the assessment of fluid overload before and after HD.

## Conclusions

In summary, this study support the notion that the value of modern biomarkers in patients with several influences on the excretion or elimination must be interpreted with caution with regards of possible fluid overload or the increased cardiovascular risk and sequential measurements might be necessary. However, standardized echocardiographic examination for the exclusion of cardiac illnesses or heart failure is the gold standard in the assessment of the fluid status and further cardiovascular risk status. Additionally, VCID is a feasible method in the hand of well-trained investigators and should be performed in all patients together with clinical data to minimize the risk for intra- or post-HD complications as hypotonic dysregulation respectively fluid overload.

## Additional file

Additional file 1: Table S1. Comparison of parameters depending on ultrafiltration rate (UF). **Table S2.** Comparison of parameters depending on residual excretion (RE). **Table S3.** Comparison of parameters depending on post-HD renin increase or post-HD renin decrease. **Table S4.** Comparison of parameters depending on post-HD aldosterone increase or post-HD aldosterone decrease. **Table S5.** Echocardiographic parameters before and after HD. **Table S6.** Echocardiographic parameters depending on the ultrafiltration rate. **Table S7.** Echocardiographic parameters depending on the residual excretion. **Table S8.** Significant correlations (Spearman-test) between echocardiographic parameters and biochemistry parameters (−: negative correlation, +: positive correlation) before and after haemodialysis (HD). **Table S9.** Significant correlations between biochemistry parameters and percentaged VCID decrease. **Table S10.** Clinical relevance of copeptin: examples of copeptin concentrations (pmol/l); mean ± SD or median (min–max). (DOCX 48 kb)

## Abbreviations

AVP: Vasopressin
BMI: Body mass index
CKD: Chronic kidney disease
CKD5D: End stage renal disease
CRS: Chronic reno-cardiac syndrome
CT-proAVP: Copeptin
EDT: Deceleration time
HD: Haemodialysis
Hk: Haematocrit
KT/v: Haemodialysis treatment adequacy
LV: Left ventricular
MR-proANP: Mid-regional pro-atrial natriuretic peptide
NT-proBNP: N-terminal prohormone of brain natriuretic peptide
RE: Residual excretion
SPAP: Systolic pulmonary arterial pressure
TAPSE: Tricuspid annular plane systolic excursion
TDI: Tissue-Doppler imaging
UF: Ultrafiltration rate
VCID: Diameter of vena cava inferior
