# Supplementary material for: Modern peptide biomarkers and echocardiography in cardiac healthy haemodialysis patients
Source: BMC Nephrol. 2017 May 30;18:175. doi: 10.1186/s12882-017-0589-3 (PMC5450351; doi:10.1186/s12882-017-0589-3)
Supplement: Additional file 1: Table S1. — Comparison of parameters depending on ultrafiltration rate (UF). Table S2. Comparison of parameters depending on residual excretion (RE). Table S3. Comparison of parameters depending on post-HD renin increase or post-HD renin decrease. Table S4. Comparison of parameters depending on post-HD aldosterone increase or post-HD aldosterone decrease. Table S5. Echocardiographic parameters before and after HD. Table S6. Echocardiographic parameters depending on the ultrafiltration rate. Table S7. Echocardiographic parameters depending on the residual excretion. Table S8. Significant correlations (Spearman-test) between echocardiographic parameters and biochemistry parameters (−: negative correlation, +: positive correlation) before and after haemodialysis (HD). Table S9. Significant correlations between biochemistry parameters and percentaged VCID decrease. Table S10. Clinical relevance of copeptin: examples of copeptin concentrations (pmol/l); mean ± SD or median (min–max). (DOCX 48 kb) [file 12882_2017_589_MOESM1_ESM.docx]

**Table S1:** Comparison of parameters depending on ultrafiltration rate (UF)

**Parameters UF ≥ 2000 ml UF < 2000 ml Significance**

Months of ongoing HD 64 (13-102) 9 (3-73) p < 0.05

Weight loss (kg) 2.4 (1.7-4.3) 0.75 (0-2.6) p < 0.05

Residual excretion (ml) 250 (0-800) 875 (300-1500) p < 0.01

Kt/V 1.3 (1.1-1.8) 1.4 (1.1-2) n. s.

BMI 27.8 (22.9-35.6) 23.7 (18.4-32.2) n. s.

Sodium before HD (mmol/l) 137 (135-144) 38 (134-140) n. s.

Sodium after HD (mmol/l) 136 (133-147) 35 (132-140) n. s.

Potassium before HD (mmol/l) 5.5 (4.4-6.2) 4.9 (2-5.9) n. s.

Potassium after HD (mmol/l) 4.3 (3.6-4.8) 4.2 (3.8-4.9) n. s.

Phosphate before HD (mmol/l) 1.8 (1.3-3.3) 1.4 (0.8-2.3) n. s.

Phosphate after HD (mmol/l) 0.9 (0.6-1.1) 0.7 (04-0.9) p < 0.05

Urea before HD (mmol/l) 27.3 (20.3-36.7) 18.7 (9.9-25.4) p < 0.01

Urea after HD (mmol/l) 8.15 (5.5-14.9) 5.3 (3-12) p < 0.05

Urea reduction (%) 69.7 (57.3-81.3) 70.6 (52.8-80.8) n. s.

Osmolality before HD (mosm/kgH2O) 324 (311-338) 308 (302-335) p < 0.05

Osmolality after HD (mosm/kgH2O) 310 (292-324) 300 (291-326) n. s.

Creatinine before HD (μmol/l) 1073 (861-1481) 830 (182-1055) p < 0.01

Creatinine after HD (μmol/l) 439 (267-702) 286 (116-403) p < 0.01

Aldosterone before HD (ng/l) 225 (35-470) 75 (37-615) n. s.

Aldosterone after HD (ng/l) 189 (36-707) 40 (10-561) p < 0.05

Renin active (direct) before HD (ng/l) 78 (1.3-683) 8.7 (1-148) n. s.

Renin active (direct) after HD (ng/l) 78 (3.4-1705) 11.5 (0.6-147) n. s.

Metanephrine before HD (ng/l) 175 (75-349) 127 (54-258) n. s.

Metanephrine after HD (ng/l) 94 (32-308) 71 (37-150) n. s.

Normetanephrine before HD (ng/l) 475 (362-822) 403 (92-611) n. s.

Normetanephrine after HD (ng/l) 209 (108-328) 177 (75-238) n. s.

AVP before HD (ng/l) 8.9 (4.4-9.4) 5.1 (1-7.7) p < 0.05

AVP after HD (ng/l) 6.9 (2.9-9.1) 2.7 (1-6.1) p < 0.01

CT-proAVP before HD (pmol/l) 207 (59-291) 90 (14-196) p < 0.05

CT-proAVP after HD (pmol/l) 110 (27-132) 29 (8-75) p < 0.01

MR-proANP before HD (pmol/l) 814 (285-1273) 793 (384-2908) n. s.

MR-proANP after HD (pmol/l) 702 (222-1100) 556 (265-2144) n. s.

NT-proBNP (ng/l) 2617 (812-17220) 3843 (522-72312) n. s.

Abbreviations: HD haemodialysis, AVP anti-diuretic hormone/vasopressin, CT-proAVP carboxy terminal pro-arginin-vasopressin (copeptin), MR-proANP midregional fragment of the N-terminal of pro-ANP, NT-proBNP N-terminal pro-brain natriuretic peptide, BMI body mass index, K dialyzer clearance of urea, t dialysis time, V volume of distribution of urea approximately equal to patient's total body water, n.s. statistically non-significant, p significance level in Mann-Whitney-U test

**Table S2:** Comparison of parameters depending on residual excretion (RE)

**Parameters RE < 500 ml n=9 RE ≥ 500 ml Significance**

Months of ongoing HD 13 (3-59) 67 (8-102) p < 0.01

Weight loss (kg) 1.1 (0-2.6) 2.2 (1.7-4.3) n. s.

Ultrafiltration rate (ml) 1300 (0-3000) 2500 (1400-5000) p < 0.05

Kt/V 1.26 (1.14-1.45) 1.5 (1.1-2.03) n. s.

BMI 27.8 (18.4-35.6) 25.6 (22.3-29.5) n. s.

Sodium before HD (mmol/l) 136 (133-147) 137 (135-142) n. s.

Sodium after HD (mmol/l) 137 (132-149) 136 (134-139) n. s.

Potassium before HD (mmol/l) 4.9 (1.9-6.2) 5.6 (4.4-6.2) n. s.

Potassium after HD (mmol/l) 4.5 (3.8-4.9) 4.1 (3.6-4.8) n. s.

Phosphate before HD (mmol/l) 1.5 (0.8-2.6) 1.8 (1.2-3.3) n. s.

Phosphate after HD (mmol/l) 0.8 (1.2-3.3) 0.9 (0.6-1.1) n. s.

Urea before HD (mmol/l) 20.8 (9.9-28) 23.6 (15.2-36.7) n. s.

Urea after HD (mmol/l) 6.4 (3-12) 6.2 (3.4-14.9) n. s.

Urea reduction (%) 66.8 (76-53) 74 (81-57) n. s.

Osmolality before HD (mosm/kgH2O) 317 (302-335) 324 (309-338) n. s.

Osmolality after HD (mosm/kgH2O) 300 (291-316) 309 (292-324) n. s.

Creatinine before HD (μmol/l) 861 (182-1055) 1109 (761-1481) p < 0.05

Creatinine after HD (μmol/l) 330 (116-439) 407 (220-702) n. s.

Aldosterone before HD (ng/l) 87 (37-453) 220 (35-615) n. s.

Aldosterone after HD (ng/l) 47 (10-707) 188 (12-561) n. s.

Renin active (direct) before HD (ng/l)9.2 (1-202) 14 (1.3-683) n. s.

Renin active (direct) after HD (ng/l) 16 (0.6-197) 32 (1.5-1705) n. s.

Metanephrine before HD (ng/l) 144 (54-349) 147 (75-258) n. s.

Metanephrine after HD (ng/l) 85 (37-150) 77 (32-308) n. s.

Normetanephrine before HD (ng/l) 389 (92-611) 575 (374-822) p < 0.05

Normetanephrine after HD (ng/l) 180 (75-238) 218 (108-328) n. s.

AVP before HD (ng/l) 5.6 (1-9.2) 7.6 (4.4-9.4) n. s.

AVP after HD (ng/l) 4.5 (1-7.2) 5.1 (2.8-9.1) n. s.

CT-proAVP before HD (pmol/l) 193 (59-291) 95 (14-218) p < 0.05

CT-proAVP after HD (pmol/l) 106 (27-132) 39 (8-114) p < 0.05

MR-proANP before HD (pmol/l) 669 (285-2908) 1027 (360-1361) n. s.

MR-proANP after HD (pmol/l) 466 (222-2144) 732 (240-1100) n. s.

NT-proBNP (ng/l) 1230 (522-72312) 3642 (1014-17220) n. s.

Abbreviations: HD haemodialysis, AVP anti-diuretic hormone/vasopressin, CT-proAVP carboxy terminal pro-arginin-vasopressin (copeptin), MR-proANP midregional fragment of the N-terminal of pro-ANP, NT-proBNP N-terminal pro-brain natriuretic peptide, BMI body mass index, K dialyzer clearance of urea, t dialysis time, V volume of distribution of urea approximately equal to patient's total body water, n.s. statistically non-significant, p significance level in Mann-Whitney-U test

**Table S3:** Comparison of parameters depending on post-HD renin increase or post-HD renin decrease

**Renin increase Renin decrease**

**(n**  **= 13) (n = 7) Significance**

Aldosterone before HD (ng/l) 230 (35-615) 57 (37-220) p < 0.05

Aldosterone after HD (ng/l) 189 (35-707) 24 (10-73) p < 0.01

Metanephrine before HD (ng/l) 216 (54-349) 120 (75-218) n. s.

Metanephrine after HD (ng/l) 87 (60-308) 62 (32-86) p < 0.01

Normetanephrine before HD (ng/l) 471 (92-822) 389 (290-539) n. s.

Normetanephrine after HD (ng/l) 205 (110-328) 168 (75-218) n. s.

AVP before HD (ng/l) 7.6 (1.9-9.4) 6.2 (1-9.2) n. s.

AVP after HD (ng/l) 5.9 (1-9.1) 3.2 (1-7.2) n. s.

CT-proAVP before HD (pmol/l) 174 (20-291) 139 (14-218) n. s.

CT-proAVP after HD (pmol/l) 80 (10-132) 39 (8-114) n. s.

Ultrafiltration rate 2500 (1000-5000) 1400 (0-2700) n. s.

There was a significant renin increase in thirteen patients after HD (p < 0.01, Wilcoxon-test; renin before HD: median 9.2 ng/l (range 1.3 to 683 ng/l); renin after HD: median 20 ng/l (range 2 to 1705 ng/l)) and a significant renin decrease in seven patients after HD (p < 0.05, Wilcoxon-test; renin before HD: median 18 ng/l (range 1 to 202 ng/l); renin after HD: median 16 ng/l (range 0.6 to 147 ng/l)).

Abbreviations: HD haemodialysis, AVP anti-diuretic hormone/vasopressin, CT-proAVP carboxy terminal pro-arginin-vasopressin (copeptin), n.s. statistically non-significant, p significance level in Mann-Whitney-U test

**Table S4:** Comparison of parameters depending on post-HD aldosterone increase or post-HD aldosterone decrease

**Aldosterone Aldosterone**

**increase decrease**

**(n = 5) (n = 15) Significance**

Haemoglobin (Hb) before HD (g/dl) 7.5 (6.7-8.2) 7.1 (5.5-8.1) n. s.

Haemoglobin (Hb) after HD (g/dl) 8.5 (7.5-9.1) 7.1 (5.5-8.4) p < 0.01

Haematokrit (Hk) before HD (%) 38 (35-41) 36 (28-40) n. s.

Haematokrit (Hk) after HD (%) 42 (38-44) 35 (27-41) p < 0.01

Metanephrine before HD (ng/l) 237 (54-349) 135 (75-258) n. s.

Metanephrine after HD (ng/l) 141 (87-147) 71 (32-308) p < 0.05

AVP before HD (ng/l) 5.6 (3-9.2) 7.6 (1-9.4) n. s.

AVP after HD (ng/l) 6.8 (1-7.6) 4.5 (1-9.1) n. s.

CT-proAVP before HD (pmol/l) 163 (20-207) 159 (14-291) n. s.

CT-proAVP after HD (pmol/l) 95 (10-119) 45 (8-132) n. s.

Ultrafiltration rate 2700 (1300-4100) 1600 (0-5000) n. s.

Systolic RR3 (mmHg) 110 (85-125) 130 (90-190) p < 0.05

Systolic RR4 (mmHg) 100 (72-125) 135 (85-160) n. s.

Abbreviations: HD haemodialysis, AVP anti-diuretic hormone/vasopressin, CT-proAVP carboxy terminal pro-arginin-vasopressin (copeptin), RR3 = systolic blood pressure two hours after begin of HD, RR4 = systolic blood pressure shortly after HD, n.s. statistically non-significant, p significance level in Mann-Whitney-U test

**Table S5**: Echocardiographic parameters before and after HD

**Parameters Before HD After HD Significance**

**median (min - max) median (min - max) (Wilcoxon-test)**

Emax (cm/s) 68 (37-141) 48 (26-144) n. s.

Amax (cm/s) 77 (31-132) 42 (24-110) p < 0.05

E/A-ratio 1.01 (0.64-1.96) 0.8 (0.46-2.47) p < 0.01

EDT (ms) 203 (166-285) 211 (84-286) n. s.

Vmax E' inferoseptal

(cm/s) 8.5 (4-21) 7.5 (4-12) n. s.

Vmax E' lateral (cm/s) 11 (7-16) 9 (5-16) p < 0.05

E/E' inferoseptal 8.1 (3.2-23.7) 6.7 (3-18) n. s.

E/E' lateral 6.4 (3.4-20.3) 5.8 (3-28.8) n. s.

VCID (mm) 17 (11-23) 13 (7-18) p < 0.01

Abbreviations: Emax maximum early-diastolic velocity of the mitral inflow, Amax maximum late-diastolic velocity of the mitral inflow, EDT Deceleration time of the E-wave, Vmax E' inferoseptal maximum early-diastolic tissue velocity in region of the basal inferoseptal segment of the left ventricle, Vmax E' lateral maximum early-diastolic tissue velocity in region of the basal lateral segment of the left ventricle, VCID Vena cava inferior diameter (during exspiration), n.s. statistically non-significant, p < 0.05 statistically significant (Wilcoxon-test)

**Table S6:** Echocardiographic parameters depending on the ultrafiltration rate

**Parameters UF ≥ 2000 ml UF < 2000 ml**

(n = 8) (n = 7)

Emax (cm/s) before HD 59 (38-77) 83 (37-142)

Emax (cm/s) after HD 46 (26-68) 53 (41-144)

Amax (cm/s) before HD 67 (31-112) 81 (60-132)

Amax (cm/s) after HD 41 (24-93) 42 (25-110)

E/A before HD 1.04 (0.7-1.67) 0.97 (0.64-1.96)

E/A after HD 0.72 (0.46-2.47) 0.87 (0.57-1.53)

EDT before HD (ms) 199 (166-214) 219 (172-285)

EDT after HD (ms) 216 (84-266) 211 (147-286)

Vmax E' inferoseptal before HD (cm/s) 9 (4-21) 8.5 (6-10)

Vmax E' inferoseptal after HD (cm/s) 6.5 (4-12) 8.5 (6-11)

Vmax E' lateral before HD (cm/s) 10.5 (7-13) 11.5 (7-16)

Vmax E' lateral after HD (cm/s) 8 (5-12) 10 (5-16)

E/E' inferoseptal before HD 6.2 (3.2-10.8) 9.4 (5.3-23.7)

E/E' inferoseptal after HD 7.1 (3-10) 6.6 (4-18)

E/E' lateral before HD 6.2 (3.5-7.7) 7.1 (3.4-20.3)

E/E' lateral after HD 6.2 (3-8.8) 5.4 (3.7-28.8)

VCID before HD (mm) 19 (13-23) 15 (11-21)

VCID after HD (mm) 13 (7-18) 12 (8-12)

Abbreviations: Emax= maximum early-diastolic velocity of the mitral inflow, Amax= maximum late-diastolic velocity of the mitral inflow, EDT= Deceleration time of the E-wave, Vmax E' inferoseptal= maximum early-diastolic tissue velocity in region of the basal inferoseptal segment of the left ventricle, Vmax E' lateral= maximum early-diastolic tissue velocity in region of the basal lateral segment of the left ventricle, VCID= Vena cava inferior diameter (during exspiration)

**Table S7:** Echocardiographic parameters depending on the residual excretion

**Parameters RA < 500 ml RA ≥ 500 ml**

(n = 8) (n = 7)

Emax (cm/s) before HD 59 (37-72) 83 (38-142)

Emax (cm/s) after HD 44 (26-62) 53 (44-144)

Amax (cm/s) before HD 68 (31-112) 81 (60-132)

Amax (cm/s) after HD 38 (24-52) 56 (31-110)

E/A before HD 0.87 (0.61-1.53) 0.91 (0.71-1.96)

E/A after HD 0.69 (0.46-2.47) 1.04 (0.64-1.67)

EDT before HD (ms) 205 (166-261) 202 (172-285)

EDT after HD (ms) 185 (84-249) 227 (147-286)

Vmax E' inferoseptal before HD (cm/s) 7 (4-21) 9 (6-11)

Vmax E' inferoseptal after HD (cm/s) 6 (4-12) 9 (5-11)

Vmax E' lateral before HD (cm/s) 11 (7-13) 11 (7-16)

Vmax E' lateral after HD (cm/s) 7 (5-12) 9 (5-16)

E/E' inferoseptal before HD 5.9 (3.2-10.8) 8.3 (3.5-23.7)

E/E' inferoseptal after HD 6.5 (3-8.8) 7.6 (4-18)

E/E' lateral before HD 5.9 (3.4-7.7) 6.6 (3.5-20.3)

E/E' lateral after HD 5.6 (3-8.8) 5.9 (4.9-28.8)

VCID before HD (mm) 14 (11-23) 18 (12-22)

VCID after HD (mm) 10 (7-18) 14 (8-17)

Abbreviations: Emax= maximum early-diastolic velocity of the mitral inflow, Amax= maximum late-diastolic velocity of the mitral inflow, EDT= Deceleration time of the E-wave, Vmax E' inferoseptal= maximum early-diastolic tissue velocity in region of the basal inferoseptal segment of the left ventricle, Vmax E' lateral= maximum early-diastolic tissue velocity in region of the basal lateral segment of the left ventricle, VCID= Vena cava inferior diameter (during exspiration), HD haemodialysis

**Table S8:** Significant correlations (Spearman-test) between echocardiographic parameters and biochemistry parameters (-: negative correlation, +: positive correlation) before and after haemodialysis (HD)

**Biochemistry Parameters Echocardiographic parameters Correlation, Significance**

Copeptin before HD - Amax before HD r = -0.5, p < 0.05

Copeptin after HD - Amax before HD r = -0.6, p < 0.05

Copeptin before HD - E/E' inferoseptal before HD r = -0.6, p < 0.05

Copeptin after HD - E/E' inferoseptal before HD r = -0.7, p < 0.01

Copeptin after HD - E/E' lateral before HD r = -0.5, p < 0.05

Renin before HD + E/E' lateral after HD r = 0.7, p < 0.01

Renin after HD + E/E' lateral after HD r = 0.6, p < 0.05

Abbreviations: Emax= maximum early-diastolic velocity of the mitral inflow, Amax= maximum late-diastolic velocity of the mitral inflow, EDT= Deceleration time of the E-wave, Vmax E' inferoseptal= maximum early-diastolic tissue velocity in region of the basal inferoseptal segment of the left ventricle, Vmax E' lateral= maximum early-diastolic tissue velocity in region of the basal lateral segment of the left ventricle, VCID= Vena cava inferior diameter (during exspiration), p < 0.05 statistically significant (Spearman-test), r regression coefficient, HD haemodialysis

**Table S9:** Significant correlations between biochemistry parameters and percentaged VCID decrease

**Biochemistry parameter Correlation, Significance**

+ Copeptin after HD r = 0.6, p < 0.05

**percentage**  + AVP before HD r = 0.7, p < 0.01

**VCID** + AVP after HD r = 0.7, p < 0.01

**decrease** + Metanephrine after HD r = 0.6, p < 0.05

+ Aldosteron after HD r = 0.5, p < 0.05

Abbreviations: VCID Vena cava inferior diameter (during exspiration), p < 0.05 statistically significant (Spearman-test), r regression coefficient, HD haemodialysis

**Table S10:** Clinical relevance of copeptin: examples of copeptin concentrations (pmol/l); mean ± SD or median (min–max)

**Clinical relevance Copeptin (pmol/l) Literature**

Coronary heart disease 43 ± 17 Chai et al., 2009

After cardiac surgery 101 ± 82 Jochberger et al., 2006

Metabolic syndrome 5.14 (3.20-8.15) Enhörning et al., 2011

Stress (after extubation) 68 (38-110) Katan et al., 2008

SIRS 88 ± 89 Jochberger et al., 2006

Sepsis 52 ± 30 Jochberger et al., 2006

Septic shock 172 (35-504) Morgenthaler et al.,2007

Haemorrhacic shock 269 (241-456) Morgenthaler et al., 2007

Chronic renal insufficiency 81 (51-122) Fenske et al., 2011

Haemodialysis patients pre-HD 159 (14-291) this publication

post-HD 63 (8-132)
